# Transcriptional analysis reveals specific niche factors and response to environmental stresses of enterohemorrhagic *Escherichia coli* O157:H7 in bovine digestive contents

**DOI:** 10.1186/s12866-021-02343-7

**Published:** 2021-10-19

**Authors:** Audrey Segura, Yolande Bertin, Alexandra Durand, Mhammed Benbakkar, Evelyne Forano

**Affiliations:** 1 Université Clermont Auvergne, INRAE, MEDIS 0454, F-63000 Clermont-Ferrand, France; 2 Université Clermont Auvergne, CNRS, IRD, OPGC, Laboratoire Magmas et Volcans, F-63000 Clermont-Ferrand, France

**Keywords:** EHEC, RNAseq, Bovine gastrointestinal tract, Stress response

## Abstract

**Background:**

Enterohemorrhagic *Escherichia coli* (EHEC) are responsible for severe diseases in humans, and the ruminant digestive tract is considered as their main reservoir. Their excretion in bovine feces leads to the contamination of foods and the environment. Thus, providing knowledge of processes used by EHEC to survive and/or develop all along the bovine gut represents a major step for strategies implementation.

**Results:**

We compared the transcriptome of the reference EHEC strain EDL933 incubated *in vitro* in triplicate samples in sterile bovine rumen, small intestine and rectum contents with that of the strain grown in an artificial medium using RNA-sequencing (RNA-seq), focusing on genes involved in stress response, adhesion systems including the LEE, iron uptake, motility and chemotaxis. We also compared expression of these genes in one digestive content relative to the others. In addition, we quantified short chain fatty acids and metal ions present in the three digestive contents. RNA-seq data first highlighted response of EHEC EDL933 to unfavorable physiochemical conditions encountered during its transit through the bovine gut lumen. Seventy-eight genes involved in stress responses including drug export, oxidative stress and acid resistance/pH adaptation were over-expressed in all the digestive contents compared with artificial medium. However, differences in stress fitness gene expression were observed depending on the digestive segment, suggesting that these differences were due to distinct physiochemical conditions in the bovine digestive contents. EHEC activated genes encoding three toxin/antitoxin systems in rumen content and many gene clusters involved in motility and chemotaxis in rectum contents. Genes involved in iron uptake and utilization were mostly down-regulated in all digestive contents compared with artificial medium, but *feo* genes were over-expressed in rumen and small intestine compared with rectum. The five *LEE* operons were more expressed in rectum than in rumen content, and *LEE1* was also more expressed in rectum than in small intestine content.

**Conclusion:**

Our results highlight various strategies that EHEC may implement to survive in the gastrointestinal environment of cattle. These data could also help defining new targets to limit EHEC O157:H7 carriage and shedding by cattle.

**Supplementary Information:**

The online version contains supplementary material available at 10.1186/s12866-021-02343-7.

## Background

Enterohemorrhagic *Escherichia coli* (EHEC) constitutes a subset of Shiga-toxin producing *E. coli* (STEC) with foodborne etiology responsible for human diseases ranging from watery diarrhea to severe illnesses such as hemorrhagic colitis (HC) and hemolytic and uremic syndrome (HUS) [1]. Although more than 400 serotypes of EHEC are known, numerous epidemiological studies have shown that EHEC strains of O157:H7 serotype are the major source of large-scale outbreaks and severe complications in industrialized countries [2]. In 2011, STEC strains of O104:H4 serotype caused a foodborne outbreak that started in Germany and affected sixteen countries, causing HC, HUS and deaths [3, 4]. *E. coli* O104:H4 strains appeared different from STEC and EHEC strains, and shared virulence properties with enteroaggregative *E. coli* (EAEC) [4]. The emergence of this new atypical STEC pathogen has complexified the nomenclature of *E. coli* enteropathotypes, usually classified according to virulence factors, infection mechanisms, interaction with enterocytes, tissue tropism, symptoms and syndromes. Key events of EHEC pathogenesis in humans are associated to the ability of EHEC strains to adhere to intestinal epithelial cells and release Shiga toxins (Stx) which are essential for virulence [5, 6]. It is well documented that colonization by EHEC (i.e., of O157:H7 serotype) induces histopathological lesions called “Attaching and Effacing” lesions (AE lesions). These lesions are characterized by an intimate bacterial adherence to the cell, a destruction of intestinal microvilli and the formation of a pedestal structure [7]. Factors responsible for AE lesions are encoded by genes located on the Locus of Enterocyte Effacement (LEE). LEE encodes (i) components of a type III secretion system, (ii) the adhesin intimin encoded by the *eae* gene and (iii) intimin’s receptor, Tir, which the pathogen inserts into intestinal mucosal epithelial cells, enabling *E. coli* to adhere to the cells with the characteristic AE lesions [7]. Importantly, the transcription of LEE-associated genes is known to be extensively regulated by distinct transcriptional regulators such as Ler and specific environmental conditions (temperature, pH, iron, ammonium, calcium, bicarbonate, quorum-sensing signaling etc.) [5, 6, 8]. The other major EHEC virulence factor is the Shiga toxin Stx. Once produced in the human gut by EHEC strains, the Stx toxins translocate across the intestinal epithelium, reach the bloodstream and then their target endothelial cells, where they bind to the globotriaosylceramide-3 (Gb3) receptors. The main Stx target organs are kidneys and brain, leading to severe lesions in these organs such as the HUS [5]. The Stx-encoding genes are prophage-borne and their expression is induced by activation of the bacterial SOS response by DNA damaging agents such as antibiotics. Two variants of *stx* genes (*stx1* and *stx2*) can be found in EHEC strains. Stx2 is produced when the phage enters the lytic cycle, while Stx1 is regulated by phage cycle and an iron-regulated promoter [5]. *E. coli* O104:H4 also carry a Stx2-encoding prophage but, instead of the *eae* gene of typical EHEC, they produce aggregative adherence fimbria mediating a tight adherence to epithelial cells, and other EAEC specific virulence factors [9]. Then, *E. coli* O104:H4 is probably an EAEC which has evolved by uptake of Stx phages [4].

The gastrointestinal tract (GIT) of healthy ruminants, particularly cattle, is considered as the primary reservoir of EHEC, although STEC can be found in a wide range of vertebrates and invertebrates [10]. Up to now, O104:H4 serotypes have not been described in cattle [11]. In bovines, EHEC carriage is mostly asymptomatic because these animals lack the endothelial Stx receptor Gb3 [12], although in young calves diarrhea has been associated to the presence of STEC and AE lesions [13, 14]. EHEC human infections, particularly due to O157 and O26 serogroups, are mainly acquired by the consumption of contaminated bovine food products such as undercooked meat and unpasteurized dairy products, or contaminated fruit and vegetable products [15]. Indeed, bovine feces excreted in the environment can lead to water, fruits and vegetables contamination, mainly through manure spreading, as well as EHEC propagation within herds. Human contamination through direct contacts with ruminants or pastured meadows has also been reported [16]. Since bovines highly contribute to EHEC human infections, finding strategies to limit EHEC carriage and shedding in bovines would allow to reduce all routes of human contamination by this pathogen. Therefore, providing knowledge of the physiology of EHEC strains during their residence in the gut bovine environment is critical to implement new strategies.

Conditions required for an efficient EHEC colonization of the host animal intestine are still not completely understood. EHEC have been found in the entire animal gut, from mouth to rectum which is considered as the main site of EHEC colonization and multiplication [17, 18]. Although *E. coli* are thought to reside preferentially along the host epithelium, O157 strains have been detected both at the mucosa and in the lumen of all the digestive compartments of cattle [17]. *In vitro* studies have shown that EHEC are able to survive and/or grow in rumen, small intestine, caecum, colon and rectum contents with different patterns suggesting that factors and processes involved in EHEC colonization are different all along the bovine gut [19–22]. After ingestion, the first digestive compartment encountered by EHEC is the rumen where high short-chain fatty acid (SCFA) concentrations represent hostile environment for EHEC survival. Also, the rumen pH can be rather low, particularly after feeding with high starch diet [23]. EHEC then go to the abomasum, much more acidic. Thus, EHEC must use several acid resistance systems to successfully transit through rumen and abomasum to reach its intestinal colonization sites [24, 25]. Similar to what has been observed in human infections, EHEC produce AE lesions in the bovine GIT. Indeed, AE lesions have been observed in the small intestine, colon and rectum of naturally and experimentally infected bovines [18, 26–28] suggesting that the LEE may be important for EHEC carriage in the bovine gut.

To survive in the bovine gut, EHEC strains must also produce distant adherence systems that allow to bring the bacteria closer to the host epithelial cells and to resist against intestinal fluxes. Flagella has been shown *in vitro* to initiate EHEC colonization to rectum epithelial cells [29]. Other fimbrial structures have been shown to promote EHEC colonization in the bovine GIT. Noteworthy, the F9 fimbriae are essential for *in vivo* EHEC colonization of calves, the hemorrhagic *coli* pilus is required for adherence of EHEC O157:H7 to bovine gut explants and type I fimbriae is a contributing factor to the colonization of EHEC O26 and O118 in cattle infection model [30–32]. Otherwise, long polar fimbriae, curli, *E. coli* common pilus and sorbitol-fermenting fimbriae have been demonstrated to be important for EHEC colonization of human epithelial cells [33].

During their transit, EHEC must also compete with the resident microbiota and use several strategies for utilizing growth-limiting nutrients [34]. It is now well documented that EHEC strains use carbohydrates released from the mucus layer covering the bovine GIT as carbon source and ethanolamine included in phospholipids constituting animal and bacterial cell membranes as a nitrogen source [35, 36]. Gluconeogenic substrates such as glycerol, lactate and amino acids are also probably used by EHEC strains as nutrients for their maintenance and/or growth in the bovine GIT when mucus-derived carbohydrates are exhausted [37, 38].

In a recent study, we investigated and compared metabolic and respiratory pathways potentially used by the reference EHEC strain EDL933 in the contents of different bovine digestive segments [21]. In the present report, we analyzed RNA-seq expression profiles to predict (i) response of EHEC to potential unfavorable conditions encountered during its transit along the bovine gut lumen and (ii) adaptive factors favoring EHEC colonization and survival in the bovine GIT. This analysis was completed by qPCR quantification of gene expression. We also measured pH, SCFA and metal ions concentrations in the bovine GIT contents used for EHEC growth.

## Results

### Physiochemical conditions of the digestive contents

pH and SCFAs (acetate, propionate and butyrate) concentrations were measured in the media used for the growth of the *E. coli* reference strain EDL933 (rumen, small intestine, rectum contents and M9 minimal medium supplemented with glucose (M9-Glc)) (Table 1). These parameters were also quantified after 3h and 6 h of *E. coli* EDL933 growth.

**Table 1:** SCFA concentration and pH (± SEM) in filtered bovine digestive contents and M9-Glc before incubation (t=0) and after 3h and 6h incubation of EHEC EDL933

		Concentration (mM)		
SCFA		T= 0	T= 3 h	T= 6h
**acetate**	M9-Glc Rumen Small Intestine Rectum	0.59 (± 0.03)^**a**^ 92.50 (± 0.66)^**a**^ 13.23 (± 0.20)^**a**^ 11.15 (± 0.17)^**a**^	1.75 (± 0.10)^**a**^ - 15.40 (± 1.81)^**a**^ 12.08 (± 0.52)^**a**^	5.42 (± 0.28)^**b**^ 83.65 (± 0.56)^**b**^ 21.12 (± 0.79)^**b**^ 11.81 (± 0.30)^**a**^
**propionate**	M9-Glc Rumen Small Intestine Rectum	1.30 (± 0.05)^**a**^ 18.91 (± 0.18)^**a**^ 0.77 (± 0.09)^**a**^ 2.25 (± 0.17)^**a**^	1.30 (± 0.07)^**a**^ - 0.63 (± 0.21)^**a**^ 2.23 (± 0.04)^**a**^	1.26 (± 0.06)^**a**^ 16.92 (± 0.17)^**a**^ 4.85 (± 1.33)^**b**^ 2.10 (± 0.05)^**a**^
**butyrate**	M9-Glc Rumen Small Intestine Rectum	0.60 (± 0.02)^**a**^ 12.48 (± 0.07)^**a**^ 0.17 (± 0.09)^**a**^ 0.82 (± 0.16)^**a**^	0.66 (± 0.04)^**a**^ - 0.17 (± 0.03)^**a**^ 0.69 (± 0.04)^**a**^	0.74 (± 0.05)^**a**^ 11.06 (± 0.13)^**b**^ 0.14 (± 0.02)^**a**^ 0.62 (± 0.03)^**a**^
**pH**	M9-Glc Rumen Small Intestine Rectum	7.43 (± 0.01)^**a**^ 7.28 (± 0.18)^**a**^ 7.95 (± 0.02)^**a**^ 7.70 (± 0.07)^**a**^	7.27 (± 0.03)^**a**^ - 7.74 (± 0.14)^**a**^ 7.40 (± 0.04)^**a**^	6.86 (± 0.03)^**b**^ 7.13 (± 0.06)^**a**^ 7.27 (± 0.02)^**b**^ 7.48 (± 0.01)^**a**^

^a,b^: Within a row, means with a common letter are not statistically different (*P* < 0.05)

pH and SCFAs concentrations were measured in the incubation media (filtered rumen and small intestine contents were used without dilution, while the rectum content was diluted 1:1 with buffer, see the Materials and Methods section)

As expected, the highest concentrations of the three SCFAs were obtained in rumen contents.

The results showed a production of acetate by EHEC EDL933 in M9-Glc (~5 mM, p=0.002) and small intestine content (~8 mM, p<0.001), and a consumption of acetate in rumen contents (~9 mM, *p*<0.001), after 6h incubation. The pH slightly decreased at the end of incubation in M9-Glc (*p*=0.001) and small intestine content (p<0.001).

We also quantified metals and heavy metals in the DCs used for growth of EHEC cells (Table 2). The concentrations of essential trace elements (Co, Cr, Cu, Ni and Zn) and toxic elements (Cd and Pb) were very low in the three DCs, the highest concentration was found for Zn in the small intestine content (~4 μg/L).

**Table 2:** Metal concentrations measured in the filtered bovine digestive contents used to incubate EHEC EDL933

	Metal mean concentration in the contents ± SEM (mg/Kg)		
Metal	rumen	Small intestine^a^	rectum^b^
Co	0.006 ± 0.001	0.018	0.016 ± 0.02
Cr	0.012 ± 0.002	0.015	0.004 ± 0.001
Ni	0.004 ± 0.001	0.031	0.046 ± 0.002
Cu	0.071 ± 0.039	0.376	0.117 ± 0.044
Zn	0.159 ± 0.004	4.139	0.426 ± 0.339
Cd	0.012 ± 0.015	0.005	0.004 ± 0.0004
Pb	0.118 ± 0.015	0.227	0.200 ± 0.036

^a^: only one sample of small intestine content was available for metal quantifications

^b^: rectum content was diluted 1:1 with buffer

The concentrations were those measured in the DCs used for incubation

### EDL933 Transcriptome Profiling

The transcriptome of the EHEC strain EDL933 grown in rumen, small intestine, rectum contents and M9-Glc was obtained by RNA-seq (SRA accession SRP136076) [21]. The EDL933 RNA samples were collected during the mid- and late-exponential growth phases (3h and 6h of incubation, respectively) in small intestine and rectum contents, and after 6h of incubation in rumen content. In this report, we analyzed the expression of genes encoding stress resistance systems and niche factors (adhesion systems, iron uptake, motility and chemotaxis) in EHEC EDL933 grown in these three digestive contents (DCs) compared to that of the bacterium incubated in M9-Glc. Comparison with M9-Glc allowed to identify the genes differentially regulated in the three DCs compared with an artificial culture medium, underlining the adaptation of EDL933 to the bovine GIT.

### Response to DC conditions

**Fig. 1:**
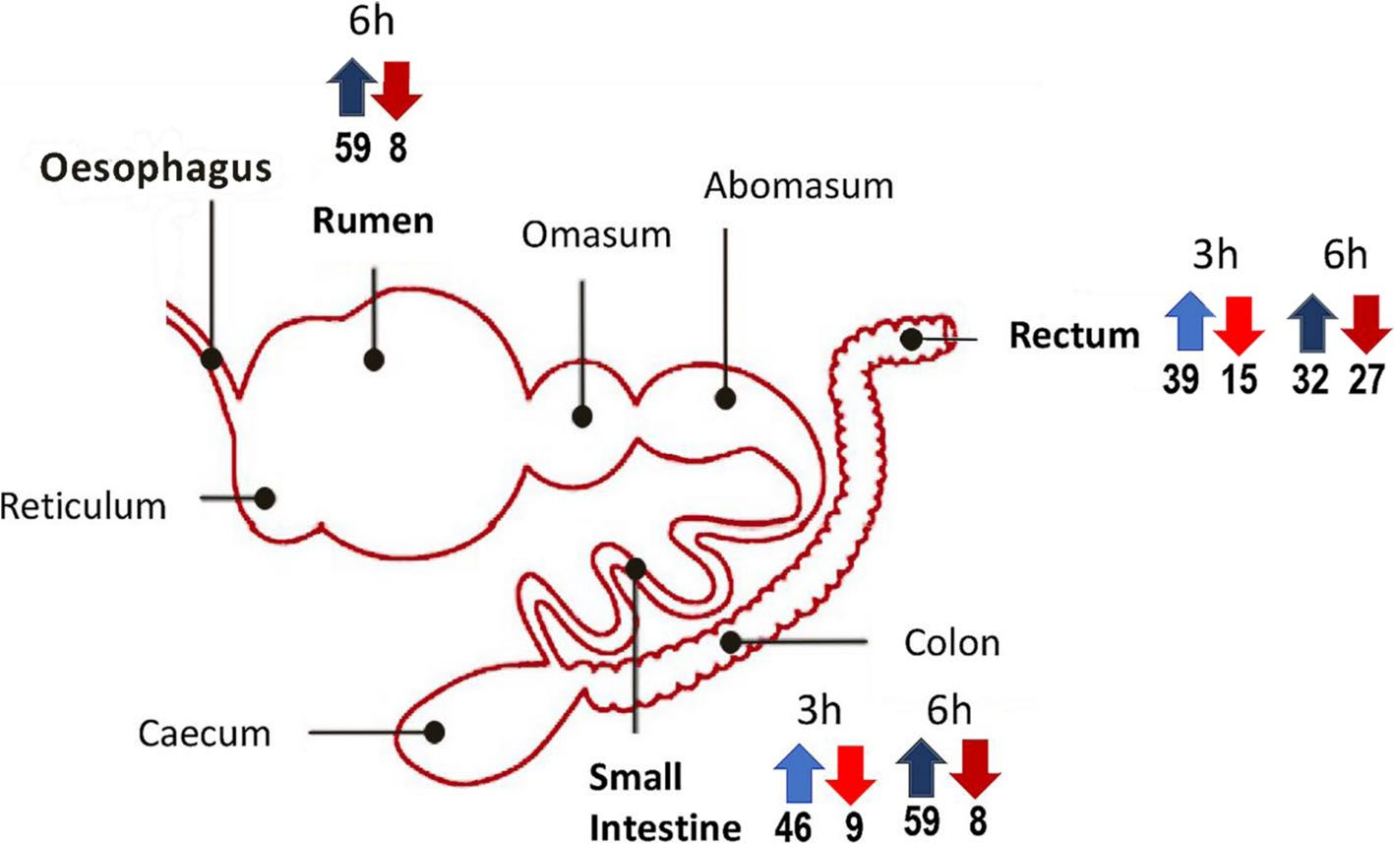
Up- and down-regulated stress-responsive genes in the three digestive contents compared with M9-Glc. The figure represents the cow gastrointestinal tract with the three digestive segments from which contents have been sampled for this study in bold. The number of EDL933 stress-responsive genes that were up- (blue arrow) or down- (red arrow) regulated after 3h (light color) or 6h (dark color) incubation are indicated below the arrows.

A total of 111 stress-related genes were found differentially expressed (Log2 FC > │2│, *q*-value < 0.05). Among them, 78 and 33 genes were found up- and down-regulated respectively after incubation in the three DCs whatever the growth phase (Additional file 1, Table S1 and S2). The number of stress-responsive genes up- and down-regulated in the three digestive contents are presented in Figure 1. The numbers of over-expressed genes compared with minimal medium are particularly high in rumen and small intestine contents. The results suggest that the conditions encountered by EHEC EDL933 in the bovine DCs appear more demanding than those found in M9-Glc minimal medium, particularly in rumen and small intestine contents (Figure 1).

**Fig. 2:**
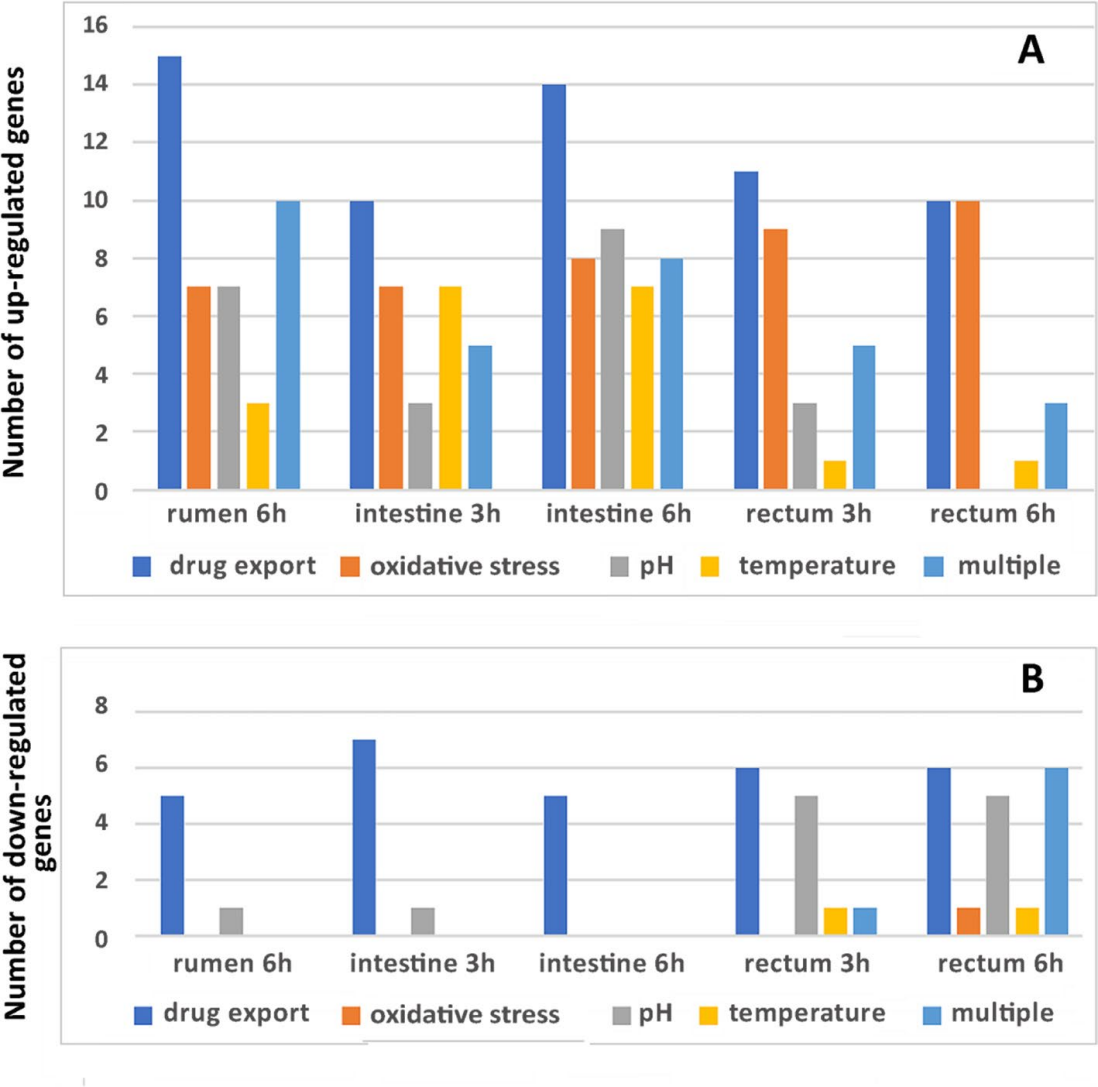
Number of the main stress-responsive genes up- (**A**) and down- (**B**) regulated in EHEC EDL933 incubated in DCs for 3h and 6h compared with M9-Glc.

Altogether, the over-expressed genes were associated with drug export (n=31), oxidative stress (n=16), acid resistance/pH adaptation (n=16), temperature change (n=10), toxin/antitoxin (TA) systems (n=5), bacterial resistance against osmotic stress (n=4) and starvation adaptation (n=3) (Additional file 1, Table S1; Additional file 2, Figure S1). Several over-expressed genes were involved in bacterial general stress response (response against multiple stresses, n=5), and/or associated to several specific stresses (n=7) (Additional file 1, Table S1). Remarkably, 8 of the 16 up-regulated genes associated with resistance against oxidative stress were over-expressed by EHEC in the three DCs during both growth phases (Additional file 1, Table S1). Also, 11 genes functionally associated with response to cadmium were over-expressed in the three DCs. However, several genes associated to specific stress response appeared more up-regulated in definite bovine compartments. The number of genes up- and down-regulated in the DCs and involved in drug export, response to oxidative stress, pH and temperature changes and response to multiple stresses are presented in Figure 2. In the rumen, 15 of the 26 genes (58%) functionally associated with drug export exhibited increased expression (Log2FC values are given in Additional file 1, Table S1). Also, 10 of the 12 genes (83%) involved in general and multiple stress response were over-expressed in the rumen content (Figure 2). Remarkably, the transcription of genes encoding three distinct TA systems was induced during incubation in the rumen (Additional file 1, Table S1). Only 5 genes related to drug export were down-regulated in this DC (Figure 2).

Regarding the small intestine, the genes associated with temperature-change response were mainly up-regulated in this compartment. An increase in expression of several genes involved in acid resistance and drug export responses was also observed in this content, as well as down-regulation of several genes related to drug-export (Figure 2 and Additional file 1, Table S1 and S2).

In the rectum content, most of the genes associated with oxidative stress were up-regulated and displayed high values of fold-change (Log2FC up to 5, Additional file 1, Table S1). Also, only a few genes involved in acid resistance response were up-regulated in rectum contents while five of them were down-regulated in this DC (Figure 2). Finally, 6 of the 11 (55%) cadmium-responsive genes exhibited increased expression in EHEC EDL933 during the mid-exponential growth phase in rectum content (Additional file 1, Table S1).

A statistical analysis with the Wilks' G^2^ independency test on contingency tables indicated that the up-regulation of the different stress gene categories in the three DCs was not dependent (p=0.112).

### Iron Uptake and Utilization

Fifty-two genes associated with uptake or utilization of iron were found differentially expressed in EHEC EDL933 during incubation in the bovine DCs compared to M9-Glc. Most of the genes (70%) were down-regulated and only 17 genes were found up-regulated in at least one of the three bovine DCs compared to M9-Glc (Additional file 1, Table S3). The down-regulated genes include genes involved in iron uptake and belonging to the *chu* operon, *efeUOB* and enterobactin gene clusters, as well as Fep genes encoding ferric Enterobactin transport [39]. Genes involved in the transport of hemin were also down-regulated. Most of these genes were down-regulated in small intestine and rectum contents at the end of the exponential growth phase compared with M9-Glc (Additional file 1, Table S3). Among the few up-regulated genes are found genes from the *suf* operon (involved in Fe-S clusters’ synthesis) and the genes *afuB* (ferric ion permease gene) and *hemH* (ferrochelatase gene) (Additional file 1, Table S3). The transcription of the *suf* operon was mainly induced in EHEC incubated for 3h (mid-exponential growth phase) in rectum contents, and the genes *afuB* and *hemH* were also over-expressed in small intestine and rectum contents at the same growth phase (3h). The *feo* genes, encoding Fe^++^ transport, were mainly induced in rumen and small intestine contents after 6h of incubation compared with M9-Glc (Additional file 1, Table S3).

We also compared directly the expression of all these genes in the different DC, to identify differential regulation from one DC to another (Additional file 1, Table S3). The results showed that the great majority of these genes were not differentially expressed in rectum *vs* rumen or rectum *vs* small intestine, except *feo* genes which appeared more expressed in rumen and small intestine contents than in rectum content.

### Chemotaxis and Motility

**Fig. 3:**
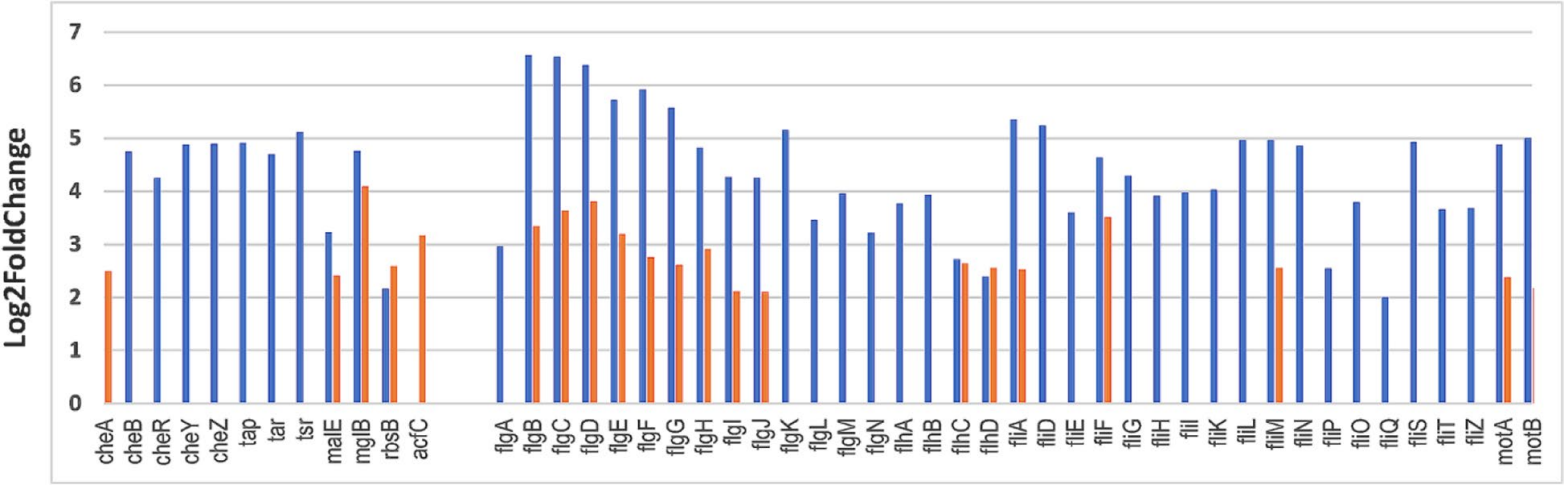
*E. coli* EDL933 chemotaxis and motility genes up-regulated after 3h (blue) or 6h (orange) of growth in rectum content compared with M9-Glc. Only genes with Log2FoldChange >2 and a Benjamini-Hochberg adjusted *p*-value (*q*-value) < 0.05 were considered as differentially expressed and reported in the Figure.

As shown in Figure 3 and in Additional file 1 Table S4, nearly all (48/49, 98%) of the genes associated to flagella synthesis and motility (37/37, 100%), and chemotaxis (11/12, 92%), were significantly over-expressed in rectum content after 3h of incubation, and 21 of them (~44%) after 6h of incubation, compared with M9-Glc. Eighteen of them were also up-regulated in small intestine after 3h of incubation. In addition, the flagellum synthesis encoding genes (*flg*, *flh*, *fli* clusters) and *mot* genes were expressed at much higher levels in EDL933 incubated in rectum content (up to 6.6 Log2FC increase in expression vs M9-Glc) than in small intestine content (Additional file 1, Table S4). None of these clusters were up-regulated in rumen contents compared with M9-Glc (except *flhC*, encoding a regulator).

The *che* genes, encoding proteins involved in chemotaxis (Additional file 2, Figure S2), were up-regulated in small intestine content (late-exponential growth phase) and rectum content (mid-exponential growth phase) compared with M9-Glc (Additional file 1, Table S4 and Figure 3). The transcription of *tsr*, *tar* and *tap*, encoding methyl-accepting chemotaxis proteins (MCPs) also involved in chemotaxis, and *malE* (encoding a maltose/maltodextrin transporter) was mainly induced in EHEC EDL933 at the mid-exponential growth phase in rectum content. Finally, the gene *acfC*, encoding an intestinal colonization factor also involved in chemotaxis, was the only one exhibiting increased expression during incubation in rumen content (Additional file 1, Table S4). Statistical analysis indicated that up-regulations of chemotaxis and motility genes were not dependent (Fisher exact test, p=0.090).

Direct comparison of gene expression in one DC *vs* the others led to similar conclusion, with both chemotaxis and motility genes being up-regulated in rectum content compared with the content of the other digestive segments, particularly after 3h incubation (Additional file 1, Table S4).

### Adhesion Systems

Transcription of the *LEE* (Locus of Enterocyte Effacement) pathogenicity island (PAI) that comprises 41 genes was analyzed (Additional file 1, Table S5).

Most of the LEE genes were down-regulated or not differentially expressed in the three DCs, compared with M9-Glc. Down-regulated genes belonged to the 5 *LEE* operons, including the type three secretion system (T3SS)-encoding genes (*LEE2* and *LEE3*), the genes coding for intimin and its receptor Tir (*LEE5*) and the genes coding for proteins involved in protein translation by the T3SS (*LEE4*) (Additional file 1, Table S5). *LEE* 3,4 and 5 genes were down-regulated in all DCs except in rectum at 6h where the genes were not differentially expressed. Genes from *LEE2* were down-regulated in rumen and small intestine contents (at 3h), while the *LEE1* genes were down-regulated only in small intestine after 3h incubation. In the other DCs, the expression was not significantly different from M9. Five *LEE* genes were found up-regulated, all at the end of the exponential growth phase in rectum content (Additional file 1, Table S5). Known *LEE*-regulators located outside the PAI were also found up-regulated in all intestine and rectum contents compared with M9-Glc. The up-regulated genes include the gene encoding the master regulator Ler.

**Fig. 4:**
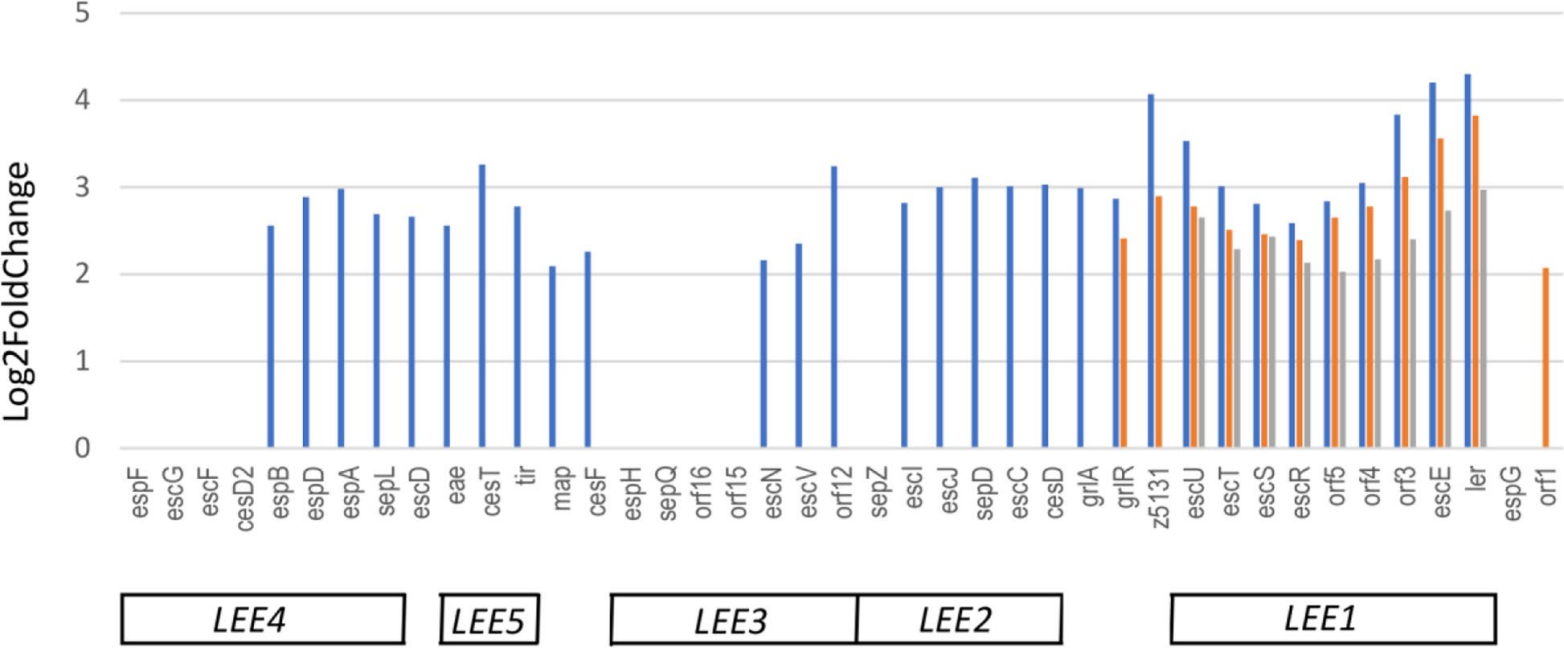
*E. coli* EDL933 LEE genes up-regulated in rectum vs rumen (6h) (blue bars), rectum vs small intestine (6h) (orange bars) and rectum vs small intestine (3h) (grey bars). Only genes with Log2FoldChange >2 and a Benjamini-Hochberg adjusted *p*-value (*q*-value) < 0.05 were considered as differentially expressed and reported in the Figure.

We also compared directly the expression of the *LEE* genes in the different DC, to confirm differential regulation from one DC to another (Additional file 1, Table S5). Direct comparison showed that nearly all of the LEE genes were up-regulated in rectum compared with rumen content (Figure 4). However, only the *LEE1* was up-regulated in rectum content compared with small intestine content.

Most of the genes encoding systems required for more distant adhesion of the bacteria (curli, fimbriae and pili) previously identified in the EDL933 genome were also either down-regulated or not differentially expressed in EDL933 incubated in bovine DCs, compared with M9-Glc (Additional file 1, Table S6). Direct comparison of gene expression in one DC *vs* the others also showed either no differential expression or down-regulation of *fim* genes in rectum compared with small intestine (3h incubation). Only some curli genes were more expressed in small intestine than in rectum, at 6h incubation (Additional file 1, Table S6).

In contrast and as already mentioned, the transcription of the gene coding for the AcfC colonization factor was induced in EHEC incubated in rumen and rectum (Additional file 1, Table S6).

### RT-qPCR Quantification of the Expression of TA Encoding Genes

**Fig. 5:**
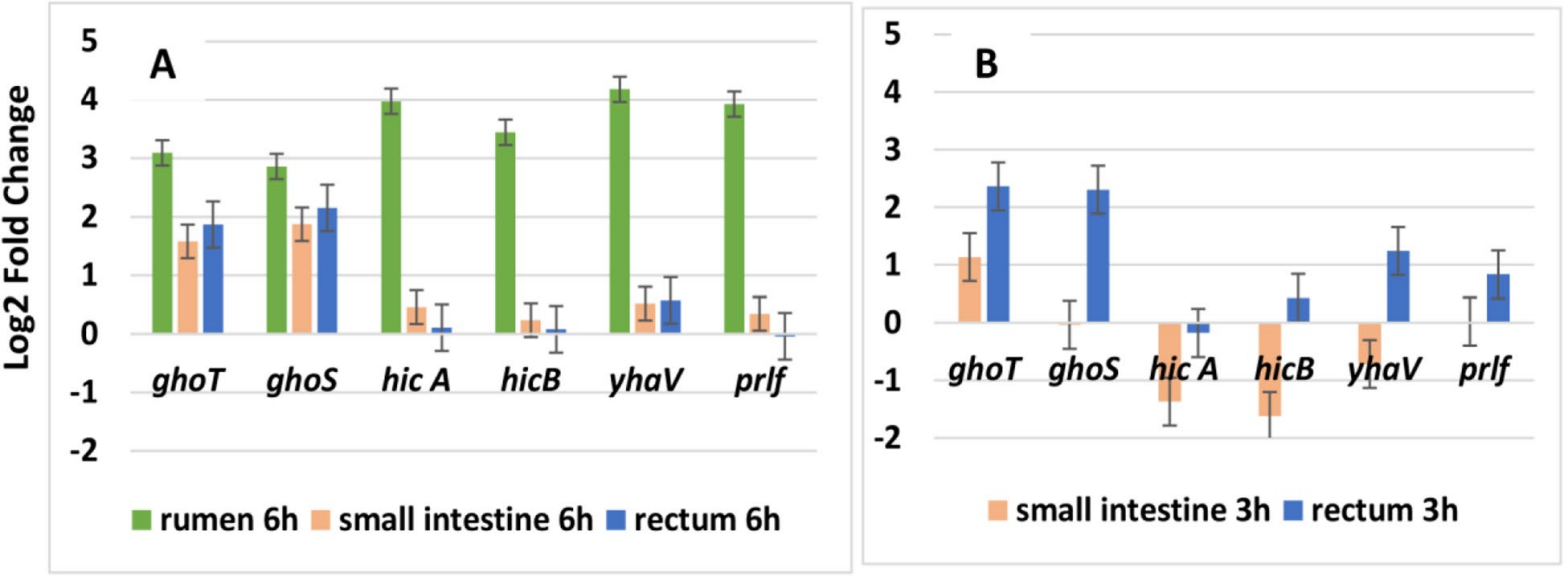
RT-qPCR quantification (Log2FC) of the expression of toxin/antitoxin encoding genes in the three DCs. Log2FC of the gene expression relative to control condition (M9-Glc) was calculated for 6h of incubation (**A**) and 3h of incubation (**B**) of EDL933 in rumen (green), small intestine (orange) or rectum (blue) contents. The presented values are the mean of the Log2FC of 4 to 6 replicates ± the standard error of the mean (SEM).

The expression of the genes coding for the three TA systems identified by RNAseq was also quantified using RT-qPCR (Figure 5). The three targeted TA systems were GhoT/GhoS, HicA/HicB, and YhaV/PrlF. After 6h incubation, the transcription of the genes encoding these three systems was clearly up-regulated (Log2FC>│2│) in rumen contents (green bars) and was significantly up-regulated in this compartment compared with small intestine and rectum (Additional file 1, Table S7). After 3h of growth, only *ghoT/ghoS* genes were found significantly up-regulated (Log2FC>│2│) in rectum content, but not in small intestine content (Figure 5). Transcription of *ghoT, ghoS, hicA* and *yhaV* genes was significantly different between small intestine and rectum after 3h incubation (p<0.05, Additional file 1, Table S7).

Finally, RT-qPCR quantification of the expression of 21 genes up-regulated in the different DCs compared with M9-Glc was also performed (Additional file 1, Table S8). These genes were included in all categories analyzed in the present work. The qPCR data correlated well, for most of them, to RNA-seq data.

## Discussion

EHEC undergo large variations in the environmental conditions during their transit through the bovine gut and need to constantly adapt to substrates and physiochemical conditions to ensure survival and/or growth. Gene expression profiles described in this report provided a broad picture of the mechanisms established by EHEC EDL933 to resist against various environmental stresses in the bovine gut lumen. Additional factors described as “Niche factors” [40] including products or strategies that probably promote the maintenance of EHEC in the bovine intestinal environment (attachment mechanisms, motility) have also been identified. In view of the results, EHEC probably encountered more unfavorable conditions in anterior digestive compartments (rumen and small intestine) than in the rectum of cattle. This is consistent with the fact that the rectum of cattle is the major site of EHEC colonization [17, 18]. In the rumen, EHEC EDL933 induced a large number of genes functionally associated with drug export, synthesis of toxin/antitoxin systems or response to acidic and oxidative stresses, compared with artificial medium. These expression patterns are reflective of the stressful rumen environment in which EHEC EDL933 is unable to multiply [19, 21, 22]. The inability of EHEC to grow in rumen contents could be due to unfavorable physiochemical conditions, but also to nutrient availability and/or inhibitory compounds released by the microbiota from the ingested plant material, such as phenolic compounds [41].

### Stress responsive genes are activated all along the GIT

The EHEC strain EDL933 activated efflux systems known to export metals, including heavy metals (cadmium, nickel, cobalt and copper), antibiotic and quaternary ammonium compounds during its growth in bovine DCs. Antibiotics are widely used in veterinary medicine, but the digestive fluids used in this study were obtained from animals which have not received any antibiotic treatment one year prior to slaughter. Industrial processing and intensive agricultural practices can result in the contamination of forage, feed and water by heavy metals, which can then be sources of exposure for farmed ruminants [42]. In this report, EHEC EDL933 induced a large number of genes functionally associated with response to cadmium in the bovine DCs, but we quantified low concentrations of Cd (from 4 to 12 μg/Kg) that cannot be considered as toxic for *E. coli* [43]. Efflux systems required to protect the bacteria against exposure to other metal ions were also activated, together with genes required to sense and face oxidative stresses. Cadmium toxicity has been associated with zinc homeostasis and oxidative stress in mammalian cells as well as in bacteria where it also depends on pH [44, 45]. The cellular redox potential depends on many parameters, but metals bearing unpaired electrons are very efficient catalysts to convert relatively inert species into highly oxidizing compounds. Reactive oxygen species (ROS) are generally detrimental to living organisms [46], and intestinal epithelial cells produce different ROS to fight against pathogenic bacteria [47, 48]. It is thus not surprising that EHEC EDL933 induced oxidative stress response in bovine gut contents. In particular, EHEC EDL933 induced the transcription of genes that are part of the regulon controlled by OxyR (*ahpCF, dps, grxA, katG, sufABCDSE, trxC* and *yaaA*). Many of these genes were up-regulated in rumen and/or rectum contents, where EHEC EDL933 was incubated under strictly anaerobic conditions. The catalase KatG and the alkyl hydroperoxide-NADPH oxidoreductase AhpCF are involved in the degradation of endogenous peroxide [49]. Dps can directly bind DNA to form a highly stable complex resistant to acid, base and oxidative stresses, and together with YaaA can sequester intracellular unincorporated iron [50–52]. The Suf proteins are also required by *E. coli* for rebuilding iron-sulfur clusters that have been oxidatively damaged [49, 53]. The expression of the gene encoding SodB, the Fe superoxide dismutase, was also increased in all the bovine DCs. Iron is an important chemical element for EHEC, and the ability of E. coli to colonize the anaerobic gut environment is specifically dependent on ferrous iron uptake [54]. Rectal administration of the iron-binding protein lactoferrin to calves experimentally infected by EHEC O157:H7 was able to reduce intestinal colonization and fecal shedding of the bacterium [55]. Different systems involved in iron uptake or utilisation were induced or repressed by EHEC during incubation in bovine DCs compared to M9-Glc: chu, efe, ent and fep clusters were down-regulated in DCs, and feo genes were the only ones clearly over-expressed in digestive contents vs M9-Glc and also in rumen and small intestine vs rectum. While Chu, Ent and Fep are mainly involved in ferric ion uptake, the Feo and Efe transporters are dedicated to ferrous iron uptake under anaerobiosis [56–59]. Feo system is required for mammal gut colonization whereas the EfeUOB transporter provides a growth advantage in minimal medium under iron-restricted conditions [54, 56, 58, 60, 61]. Accordingly, our data showed that these two transporters were activated differently in DCs and M9-Glc, and suggested that the Feo transport system has an important role in ferrous iron uptake during the transit of EHEC in bovine rumen and small intestine of cattle.

### Genes involved in acid resistance are up-regulated in rumen and small intestine contents

It is well documented that the bovine intestinal environment contains high levels of short-chain fatty acids (SCFAs) (weak acids) produced by the endogenous microbiota, particularly in the rumen, caecum, colon and rectum of cattle [21, 62]. Consequently, mechanisms that confer resistance to weak acids should contribute to EHEC survival and colonization in the bovine intestine. As expected, we measured a high concentration of acetate, propionate and butyrate in rumen contents, and lower concentrations in the other DCs. The pH in the DCs collected for this study was not acidic, due to the fact that these DCs were sampled in the morning at slaughter on animals that had been fasted since the previous day. The pH decreased significantly in small intestine content after incubation of EHEC, but did not fall below 7. However, the arginine- and lysine-dependent acid resistance (AR) systems were both activated in EHEC incubated in rumen and small intestine contents. The effectiveness of these AR systems is correlated to the pH optima of the decarboxylase enzymes [63]. The lysine-dependent AR system would enhance *E. coli* survival in mildly acidic conditions. The arginine-dependent AR systems is thought to allow *E. coli* survival in extreme low pH conditions [64], which is not the case here, but this system was also shown to be effective against a SCFA cocktail mimicking the intestinal composition [65]. Thus, EHEC EDL933 could induce this system under our experimental conditions to counteract the high concentration of organic acids in the DCs, particularly in rumen contents in which the bacterium consumes acetate. Also, *E. coli* O157:H7 Sakai was shown to over-expresses *yjiO*, a gene encoding a multidrug efflux system, only in the presence of lactic and acetic acids [66]. This gene was also up-regulated here by EDL933 in rumen contents, probably to avoid intracellular accumulation of the ionized form of the organic acids, detrimental to the bacteria [49]. Overall, the up-regulation of genes involved in acid resistance by EHEC probably results from the high organic acid concentrations in the gut contents.

### Toxin/antitoxin systems are activated in rumen content

Interestingly, EHEC EDL933 induced genes coding for components of three toxin/antitoxin (TA) systems (GhoT/GhoS, HicA/HicB and YhaV/PrlF) when incubated in rumen fluids. TA systems, involved in stress responses in prokaryotes, generally consist of i) a stable toxin, which causes bacterial growth arrest and possibly bacterial programmed cell death during stress conditions, and ii) a labile antitoxin, which sequesters the toxin into an inactive complex during normal conditions [67]. The TA systems GhoT/GhoS and HicA/HicB are known to increase bacterial resistance to chemical stresses and amino acid or glucose starvation respectively, whereas YhaV is involved in cell-cycle arrest due to nitrogen and magnesium starvation in *E. coli* [68–71]. GhoT can reduce *E. coli* metabolism by decreasing cellular ATP and proton motive force and/or lead to the formation of dormant cells under stress conditions [68, 72]. This could help EHEC to survive under rumen unfavorable conditions. We have recently demonstrated that genes encoding the GhoT/GhoS and HicA/HicB TA systems were induced in EHEC EDL933 incubated in bovine feces at 15°C [22]. Although the regulation of TA systems is very complex and can occur both at transcriptional and post-transcriptional level [67], altogether our results suggest an important role of TA systems in both EHEC colonization and persistence in the bovine gut as well as its persistence outside the animal GIT. Further work should test the ability of EHEC TA mutants to colonize the ruminant GIT.

### Flagella synthesis and chemotaxis genes are activated in rectum content

Bacteria living in a complex environment seek for and migrate to optimal environmental conditions. In this context, motility by means of flagella and chemotaxis permits the bacteria to avoid detrimental locations and to find more favorable ecological niches. This can provide EHEC with an important advantage during its transit through the bovine gut. In this report, the expression of the genes encoding the H7 flagella, known to be highly regulated by environmental stimuli [6], was strongly activated in EHEC EDL933 growing in rectum content, despite a high energetic cost. This suggested that stimuli required for flagella synthesis are present in rectum content, and not in rumen content, or are present at different concentration in the various digestive compartments [6, 73]. Indeed, it was shown previously that low concentrations of SCFAs decrease flagella gene *fliC* expression and motility [74, 75]. The H7 flagella were also previously shown to adhere to the bovine rectal epithelium [29], and *fliC* was recently found activated in EDL933 bound to cattle colonic explants [76]. Our results support the view that EHEC need functional motility when reaching the rectum of cattle, in order to attach to the rectal epithelium [6, 27]. Chemotaxis seemed also to be required by EHEC to detect and follow external stimuli in the bovine gut. Indeed, we found that the genes coding for motor switch (*fliGMN*) and rotation (*motAB*), required to rotate the flagella and move to attractant, were activated by EDL933 in rectum content. In addition, chemical attractants are directly or indirectly sensed by *E. coli* strains by means of methyl-accepting chemotaxis proteins (MCPs) [77, 78]. In this report, we highlighted three MCP-encoding genes exclusively activated by EHEC EDL933 in rectum content: the encoded proteins Tsr and Tar mediate direct chemotaxis to serine and aspartate/maltose, respectively, and Tap mediates indirect chemotaxis to dipeptides [78]. In the rectum, aspartate and serine can be released from mucus degradation [37], and maltose can be provided through intracellular glycogen degradation from the endogenous microbiota [21]. In addition, EHEC EDL933 activated a gene coding for a protein similar to the AcfC sulfate-binding protein known to enhance chemotaxis towards intestinal mucins in *Vibrio cholerae* [79, 80]. Our results support the view that EHEC EDL933 establish a gradient sensing strategy that makes cells move unidirectionally toward the highest levels of stimuli that probably favor EHEC persistence in the rectum of cattle.

### *LEE* Genes are activated in rectum content

The genes encoding the Locus of Enterocyte Effacement (LEE), responsible for the intimate attachment of EHEC to mammalian intestinal cells, were more expressed in minimal medium, completely lacking host cell related compounds, than in bovine DCs. This result is in agreement with previous work showing that LEE-encoding genes were more expressed in minimal medium than in cattle feces [81]. The LEE comprises five polycistronic operons (*LEE1-5*) regulated by numerous transcription factors in response to a large number of stimuli [82]. However, direct comparison of *LEE* gene expression in rectum *vs* rumen or small intestine contents showed that *LEE1-5* genes were more expressed in rectum than in rumen content (including *eae* and *tir* genes), and that *LEE1* (including the gene encoding the major LEE regulator *Ler*) was more expressed in rectum than in small intestine content. Anaerobiosis and the presence of NaHCO_3_ buffer in rectum content may have stimulated the production of intimin, Tir, EspA and EspB through activation of the regulator Ler, as previously shown [83]. Finally, our results agree well with many previous works showing that *eae* and *tir* play a major role in colonizing the bovine intestine [26, 28] and for enteropathogenicity of calves [13], and that the T3SS-associated proteins EspA, intimin and Tir have been used for producing vaccines which reduced colonization and shedding of EHEC O157 from experimentally infected calves [84, 85].

As for the LEE, expression of the genes encoding adhesion systems required for more distant adhesion of the bacteria to host enterocytes (Bfp [Bundle-forming pilus], Ecp [common pilus], type 1 fimbriae, curli and F9 fimbriae) was higher in EHEC EDL933 incubated in M9-Glc than in bovine DCs. Although these results do not mean that these genes are not expressed at all in DCs, they could indicate that high expression of these genes requires contact of the bacteria with the bovine epithelium, condition lacking in our experimental model. Nonetheless, comparing the expression of these adhesion genes in the various DCs showed that *acfC* and the curli *csgBAC* operon were expressed at higher level in rectum than intestinal contents. The curli fimbriae operons are expressed in response to environmental stress factors such as nutrient limitation, which probably occurs in rectum content, and curli fimbriae also help to colonize animal tissues [86]. AcfC is a chemotaxis-related protein identical to the Paa protein of porcine enteropathogenic *E. coli* that induce intimate adhesion and AE lesions in intestinal epithelial cells [87]. Further investigations are necessary to explore the role of AcfC in the adhesion of EHEC to bovine intestinal cells.

Altogether, our results suggest that EHEC EDL933 regulates the expression of the *LEE* and a few other adhesion factors in a progressive manner in the bovine GIT, increasing their transcription from the rumen to the rectum, their major site of attachment [27].

## Conclusions

In summary, this report highlighted for the first-time different stresses that EHEC strains probably face during their transit through the bovine gut lumen. Remarkably, EHEC induced numerous stress fitness genes required to neutralize toxic compounds and to resist against oxidative stresses all along the bovine gut. Toxin/antitoxin systems were induced by EHEC for successful survival in the stressful rumen environment, and niche factors involved in motility and chemotaxis as well as *LEE* genes were activated in rectum contents, the main colonization site. Taken together, our results open new avenues that will obviously require further investigations to better understand strategies used by EHEC to survive in the gastrointestinal environment of cattle, particularly in the presence of the microbiota and host cells. This could also help defining new targets and strategies to limit EHEC O157:H7 carriage and shedding by cattle.

## Materials and Methods

### Bovine Digestive Contents

Digestive contents (DCs) from rumen, small intestine and rectum compartments were collected on eight healthy “Salers” bulls from the “Herbipole” experimental Unit at the INRAE (Saint-Genès-Champanelle, France) as previously described [21]. Briefly, bulls, approximatively 2 years of age and 562 (±26) kg mean weight, were raised according to current INRAE ethical guidelines for animal welfare. These animals were fed a mixed diet composed of hay (8.6 kg /head/day) and concentrate (2.5 kg/head/day) which composition is given in Additional file 1, Table S9. The bulls had not received any antibiotic treatment in the year prior to slaughter. The bulls were slaughtered in the experimental slaughterhouse of the “Herbipole” (Permit number: 63345001). The animals had received their last meal the day before slaughter. The experiments were approved by the local ethics committee (Comité d’Ethique pour l’Expérimentation Animale en Auverggne, C2E2A, Permit Number: C6334517).

DCs from rumen and small intestine (jejunum and ileum) were collected at slaughter whereas rectum contents were collected two days before slaughter by rectal palpation. All DCs were rapidly collected and immediately brought to the laboratory. Small intestine contents were directly distributed in sterile tubes without any particular attention paid regarding anaerobiosis while rumen and rectum contents were processed under strictly anaerobic conditions as previously described [21]. Briefly, rumen contents were filtered through four layers of cheesecloth to remove large feed particles, and rectum contents were diluted 1:1 in reduced potassium phosphate buffer (50 mM potassium phosphate, resazurin 0.1%, 40 mM Na_2_CO_3_, 3 mM cysteine, pH 7.6) in order to maintain a low redox potential. Rumen and rectum samples were distributed in sterile O_2_-free CO_2_-saturated Hungate tubes (Bellco, USA). The endogenous microbiota was then removed as previously described [21]. Briefly, DCs were centrifuged twice for 15 min at 10,000 x g, and supernatants were filtered successively through membranes pore size 0.45 and 0.22 μm (Millipore). Filtrates from rumen and rectum contents were then placed in an anaerobic chamber (JACOMEX, Lyon, France) under CO_2_ atmosphere (< 80 ppm of O_2_) during three days at room temperature. After getting out of the anaerobic chamber, the samples were filtered again (0.22 μm pore-size filters, Millipore) and placed into new O_2_-free CO_2_-saturated sterile Hungate tubes. Sterility was verified on Luria Bertani (LB) agar plates after overnight incubation at 37°C. Filtered DCs were stored at 4°C until use.

### Bacterial Strain and Growth Conditions

The reference EHEC O157:H7 strain EDL933, isolated from contaminated hamburger meat [88], was used in this study. The strain was inoculated from a single colony and incubated in LB medium for 7h at 37°C with aeration. The cultures were then 50-fold diluted in filtered DCs and grown overnight at 39°C without aeration. The next day, the bacterial concentration was adjusted (OD_600 nm_) to ≈ 10^8^ bacteria mL^-1^ for inoculating rumen content and to 10^6^ bacteria mL^-1^ for inoculating small intestine and rectum contents. All DCs were incubated at 39°C (internal bovine temperature) under conditions reflecting the *in vivo* conditions for each bovine digestive compartment: i) under strict anaerobiosis with gentle shaking (rumen content) or without shaking (rectum content) or ii) under oxygen-limited conditions without shaking in small intestine content as previously described [21]. *E. coli* EDL933 was also cultured under oxygen-limited conditions in M9 medium (DIFCO) supplemented with glucose (4 g.L^−1^), MgSO_4_ (1 mM), CaCl_2_ (0.1 mM), vitamin B12 (cyanocobalamin, 150 nM), vitamin B1 (5 mg. L^−1^) and trace metals (0.1 μM ZnSO_4_, 0.045 μM FeSO_4_, 0.2 μM Na_2_Se_2_O_3_, 0.2 μM Na_2_MoO_4_, 0.1 μM MnSO_4_, 0.1 μM CuSO_4_, 0.3 μM CoCl_2_ and 0.1 μM NiSO_4_) (M9-Glc). All the experiments with EHEC O157:H7 strain were carried out in a containment laboratory in compliance with INRAE biosafety and biosecurity protocols and according to European recommendations.

### RNA Sequencing Experiments

Transcriptome analysis was performed from RNA collected after incubation of EHEC EDL933 (i) in filtered rumen after 6h of incubation, (ii) in small intestine and rectum contents after 3h and 6h of incubation and (iii) in M9 minimal medium after 3h and 6h of incubation. The M9 minimal medium was supplemented with glucose (40 mM), MgSO_4_ (1 mM), CaCl_2_ (0.1 mM) and trace metals (M9-Glc), and adjusted to ≈ pH 7.4. EHEC EDL933 was grown in M9-Glc (concentration adjusted [OD_600 nm_] to ≈ 10^7^ bacteria mL^-1^) under conditions described above for filtered small intestine content. Three biological replicates were performed for each culture condition. The bacterial counts obtained before and after incubation are given in Additional file 1, Table S10.

RNA was extracted as previously described [21], and RNA purification was performed using the Nucleospin® RNA (Macherey Nagel) according to the manufacturer recommendations. RNA quality was analyzed using an Agilent 2100 Bioanalyser (Agilent technologies, France). Samples showed 23S/16S rRNA ratio ≈ 2 and RNA Integrity Number ≥ 8. Ribodepletion was done with the MicrobExpress™ Bacterial mRNA Purification kit (Ambion), and assessed using an Agilent 2100 Bioanalyser (Agilent technologies, France).

RNA-seq libraries were prepared at the GeT-PlaGe core facility, INRAE Toulouse, France according to Illumina’s protocols as previously described [21]. Sequencing was performed on an Illumina HiSeq3000 using a paired-end read length of 2x150 bp with the Illumina HiSeq3000 chemistry.

### RNA-seq Data Analysis

After trimming to remove low quality reads and adapters (cutadapt version 1.8.3, standard parameters) [89], reads were aligned to EDL933 genome (Genbank accession numbers NZ_CP008957.1 and NZ_CP008958.1) [90] using bwa mem (version 0.7.12-r1039, standard options) [91]. Additional gene annotations from older published EDL933 chromosome and plasmid sequences were also collected (AE005174.2 [92] and AF074613.1 [93]). Additional gene annotations were also performed using the Kyoto Encyclopedia of Genes and Genomes (KEGG). Reads were counted using FeatureCount (version v1.4.5-p1) [94]. Differential gene expression was identified using DESeq2 version 1.12.4 [95] with R version 3.3.2 following the standard workflow. Fold change was calculated by comparing the expression ratio of each gene from a specific DC relative to M9-Glc. Genes with a Log2 fold-change (Log2FC) in expression greater than│2│and a Benjamini-Hochberg adjusted *p*-value (*q*-value) smaller than 0.05 were reported as differentially expressed. Differentially expressed genes were assigned to functional categories of Clusters of Orthologous Groups (COGs) of proteins using blastp against the NCBI COG 2014 database [96].

RNA-seq data have been deposited under SRA accession SRP136076.

### Reverse Transcription and Quantitative PCR (RT-qPCR)

One microgram of each RNA sample (in triplicates) was reverse transcribed using the SuperScript II Reverse Transcriptase kit (Invitrogen) with 3 μg of random primer and 100 units of SuperScript II Rnase H. The RNA samples used for retrotranscription and qPCR were the same as the ones used for RNAseq analysis. Quantitative PCR runs were carried out using the Mastercycler ep realplex apparatus (Eppendorf) using the conditions previously described [21]. The house keeping gene *mdh* was used for normalization of mRNA quantification. The relative RNA quantification was performed using primers designed to specifically amplify fragments of 90 to 200 bp (Additional file 1, Table S11). Three biological samples were used for each DC. Results were calculated using the comparative cycle threshold method. The results presented are average from two technical replicates of each biological replicate.

### pH and SCFAs Quantification

SCFAs concentration was quantified in filtered DCs and M9-Glc. A total of 30 μL of orthophosphoric acid (75%) were added to 1 mL of filtered DC and acetate, propionate and butyrate concentrations were determined by gas chromatography by AFYREN INVESTMENT (Biopole Clermont Limagne, Saint Beauzire, France). pH measurements in filtered DCs and M9-Glc were done using a HI-8424N pH meter (HANNA instruments).

### Quantification of Metal Concentrations in Bovine Digestive Contents

The concentrations of essential (cobalt, chromium, copper, nickel, and zinc) and toxic (cadmium and lead) trace elements were determined in the filtered DCs by inductively coupled plasma atomic emission spectrometer (ICP-AES). The analytical procedure for ICP-AES was as follows: five 0 to 1 ppm solutions containing the elements to analyze (Co, Cr, Ni, Cu, Zn, Cd, Pb) were prepared for calibration. An ULTIMA-C spectrometer (Horiba scientific, Jobin-Yvon) was used. This instrument combines two spectrometers to measure emission lines from elements excited in a single plasma torch: one polychromator and one scanning monochromator. Only the high-resolution scanning monochromator was used for the sequential determination of all the emission lines. The ICP-AES operating conditions were the following: incident power 1.1 kW; reflected power <15 W; plasma gas flow rate 16 L/min; permanent sheath gas flow rate 0.2 L/min; carrier gas flow rate 0.8 L/min; and solution uptake 0.9 L/min. The analytical lines used were 228.616 nm (Co), 267.716 nm (Cr), 231.604 nm (Ni), 324.754 nm (Cu), 206.2 nm (Zn), 226.502 nm (Cd), and 220.353 nm (Pb).

### Statistical Analysis

Numbers of over-expressed genes involved in chemotaxis-motility or in stress responses in the three DCs were analyzed by the Fisher exact test and the Wilks' G^2^ independency test on contingency tables, respectively, using XLSTAT.

For RT-qPCR data as well as for pH and SCFAs concentrations, analysis of variance was done using ANOVA followed by the Tuckey HSD post-hoc test for multiple means comparisons (95% family-wise confidence level). The data were analyzed using R version 4.0.3 [97].

## Supplementary Information

**Additional file 1: **Additional Tables (xlsx). **Table S1**: *E. coli* O157:H7 strain EDL933 up-regulated stress-responsive genes (RNA-seq data), **Table S2**: Down-regulated stress responsive genes, **Table S3**: Up and down-regulated genes associated with iron uptake or utilization, **Table S4**: Up-regulated genes associated with chemotaxis and flagella synthesis/motility, **Table S5**: Expression of LEE-encoding genes, **Table S6**: Expression of genes encoding proteins involved in distant adhesion of the bacteria, **Table S7**: p-values of one way ANOVA followed by Tuckey test for comparing transcription of TA systems in the three digestive contents, **Table S8**: Quantification of expression (Log2FC) of selected genes in EDL933 incubated in rumen, small intestine and rectum contents relative to M9-Glc by RT-qPCR and comparison with RNA-seq data, **Table S9**: Composition of the concentrate fed to the bulls, **TableS10**: EDL933 cell counts in the incubation media, **Table S11**: Primers used for qPCR.

**Additional file 2: Figure S1** (docx): Stress-responsive genes up-regulated in EHEC EDL933 incubated in bovine DCs during 3h (A) or 6h (B) ; **Figure S2** (docx): Pathways involved in bacterial chemotaxis

## Data Availability

RNA-seq data have been deposited under SRA accession SRP136076. All other data generated or analyzed during this study and not present in Additional File 1 are available from the corresponding author on reasonable request.
