# Supplementary material for: Transcriptional analysis reveals specific niche factors and response to environmental stresses of enterohemorrhagic Escherichia coli O157:H7 in bovine digestive contents
Source: BMC Microbiol. 2021 Oct 19;21:284. doi: 10.1186/s12866-021-02343-7 (PMC8524897; doi:10.1186/s12866-021-02343-7)
Supplement: Supplementary file 2 — Additional file 2: Figure S1 (docx): Stress-responsive genes up-regulated in EHEC EDL933 incubated in bovine DCs during 3h (A) or 6h (B) ; Figure S2 (docx): Pathways involved in bacterial chemotaxis [file 12866_2021_2343_MOESM2_ESM.docx]

**Transcriptional analysis reveals specific niche factors and response to environmental stresses of enterohemorrhagic *Escherichia coli* O157:H7 in bovine digestive contents**

**Audrey Segura^1,#^, Yolande Bertin^1^, Alexandra Durand^1^, Mhammed Benbakkar^2^ and Evelyne Forano^1,*^.**

^1^ Université Clermont Auvergne, INRAE, MEDIS 0454, F-63000 Clermont-Ferrand, France.

^2^ Université Clermont Auvergne, CNRS, IRD, OPGC, Laboratoire Magmas et Volcans, F-63000 Clermont-Ferrand, France

# present address: Chr. Hansen A/S, Bøge Allé 10-12, 2970 Hørsholm, Denmark

* Correspondence: evelyne.forano@inrae.fr

**Supplementary Figures**

**Figure S1.** Stress-responsive genes up-regulated in EHEC EDL933 incubated in bovine DCs during 3h (A) or 6h (B).


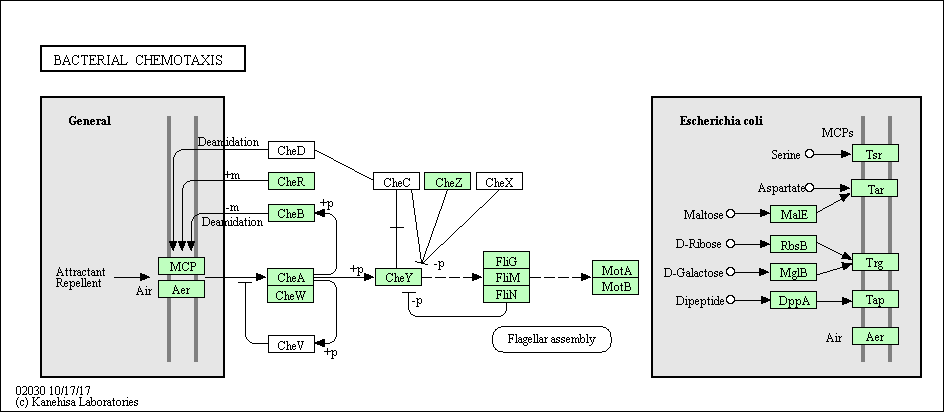


**Figure S2.** Pathway involved in bacterial chemotaxis

Green boxes indicate genes coding for proteins involved in chemotaxis (ece02030, KEGG pathway) present in the EDL933 genome.
